# Cyanidin-3-*O*-glucoside extracted from the Chinese bayberry (*Myrica rubra* Sieb. et Zucc.) alleviates antibiotic-associated diarrhea by regulating gut microbiota and down-regulating inflammatory factors in NF-κB pathway

**DOI:** 10.3389/fnut.2022.970530

**Published:** 2022-08-24

**Authors:** Yanshuai Wang, Jiebiao Chen, Yue Wang, Fanghong Zheng, Meiyu Qu, Ziwei Huang, Jialang Yan, Fangping Bao, Xian Li, Chongde Sun, Yixiong Zheng

**Affiliations:** ^1^Department of General Surgery, School of Medicine, The Fourth Affiliated Hospital, Zhejiang University, Yiwu, China; ^2^Laboratory of Fruit Quality Biology, The State Agriculture Ministry Laboratory of Horticultural Plant Growth, Development, and Quality Improvement, Fruit Science Institute, Zhejiang University, Hangzhou, China; ^3^Department of Pharmacology, School of Medicine, Zhejiang University, Hangzhou, China; ^4^Department of Anesthesiology, School of Medicine, The Fourth Affiliated Hospital, Zhejiang University, Yiwu, China

**Keywords:** antibiotic-associated diarrhea, Chinese bayberry, cyanidin-3-*O*-glucoside, gut microbiota homeostasis, intestinal tight junction protein

## Abstract

Chinese bayberry has been used to treat diarrhea in China for more than 2,000 years, but the mechanism is not clear. Due to the extensive use of antibiotics, antibiotic-associated diarrhea (AAD) is becoming more and more common in clinic, but there is no effective drug for the treatment. The present study aimed to explore the therapeutic effect of Chinese bayberry on AAD for the first time, and explained the underlying mechanism from different aspects. The BALB/c mice model was established by intragastric administration of lincomycin (3 g/kg). Successfully modeled mice were treated with purified water, dried bayberry powder suspension (100 mg/kg), C3G suspension (40 mg/kg) and montmorillonite powder suspension (40 mg/kg), respectively. The changes of body weight, diarrhea index, diarrhea status score were recorded and calculated regularly. 16S rRNA gene sequencing, intestinal immunofluorescence and inflammatory factor detection were further performed. The treatment with dried bayberry powder suspension and C3G suspension could rapidly reduce the diarrhea score and diarrhea index, increase food intake and restore body weight gain. The gut microbiota richness and diversity were significantly increased after dried bayberry powder suspension and C3G suspension treatments, typically decreased bacterial genera *Enterococcus* and *Clostridium senus stricto 1*. In addition, intake of Chinese bayberry powder and C3G significantly decreased the level of p65 phosphorylation, and up-regulated the expression of intestinal tight junction protein claudin-1 and ZO-1. Chinese bayberry fruit had the effect of alleviating AAD, and C3G was supposed to play the predominant role. The mechanism was indicated to be related with restoring the homeostasis of gut microbiota, inhibiting the level of harmful bacteria and increasing the abundance of beneficial bacteria, down-regulating TNF-α, IL-6, and IL-12 factors to reduce inflammation, restoring intestinal tight junction proteins and reducing intestinal permeability.

## Introduction

Antibiotics are widely used in the clinical treatment of various bacterial infections. Antibiotic-associated diarrhea (AAD) is a common and troublesome complication (1). AAD can be divided into two types: simple diarrhea and pseudomembranous colitis (PMC). AAD will hinder the recovery of the disease. It is reported that if left untreated, PMC with *Clostridium difficile* infection can be life-threatening, with a mortality rate of 15–24% (2). The pathogenesis of AAD is still under study. It is generally believed that antibiotics can destroy the homeostasis of gut microbiota and cause diarrhea by reducing physiological beneficial bacteria and increasing facultative anaerobes, leading to secondary infection susceptibility and eventually leading to mucosal damage (3). The human intestinal tract is occupied by a rich variety of microbial communities, including eukarya, bacteria, and archaea. And the bacteria were mainly composed of Bacteroidetes, Firmicutes, Proteobacteria, Verrucomicrobia and Actinobacteria (4). The composition and function of gut microbiota are closely related to human health and play an important role in maintaining physiological balance (5). At present, the pathogenesis of AAD is not clear, and there is a lack of effective therapeutic drugs. The research on the treatment of AAD mainly focuses on probiotics, prebiotics and natural products (6–8).

The Chinese bayberry is a characteristic fruit in China, which is mainly distributed in the south of the Yangtze River. Zhejiang Province is the major producing area of red bayberry in China, and the cultivated area, yield and production value ranks first in the national bayberry industry. This fruit can be eaten fresh and also be used to make fruit juice and wine (9). Compared with other berries, Chinese bayberry fruit is rich in cyanidin-3-*O*-glucoside (C3G), which accounts for at least 85% of anthocyanin content, and is similar to blackberry, and much higher than cranberry, blueberry, raspberry, etc., (10). The various bioactivities of bayberry fruit extracts include anti-diarrhea (11), anti-cancer (12), anti-bacteria (13, 14), antioxidant (15) and anti-diabetes (16, 17). However, due to the lack of in-depth research and scientific basis, its antidiarrheal effect has not been further investigated and developed. Up to now, there is no report on the treatment of AAD by Chinese bayberry. To further understand the biological activity of Chinese bayberry fruit and its bioactive components, this study mainly focused on the antidiarrheal effects of dried bayberry powder and C3G, through the analysis of the composition and diversity of gut microbiota, inflammatory signaling pathway and intestinal tight junction protein.

## Materials and methods

### Materials, chemicals and reagents

**Figure 1:**
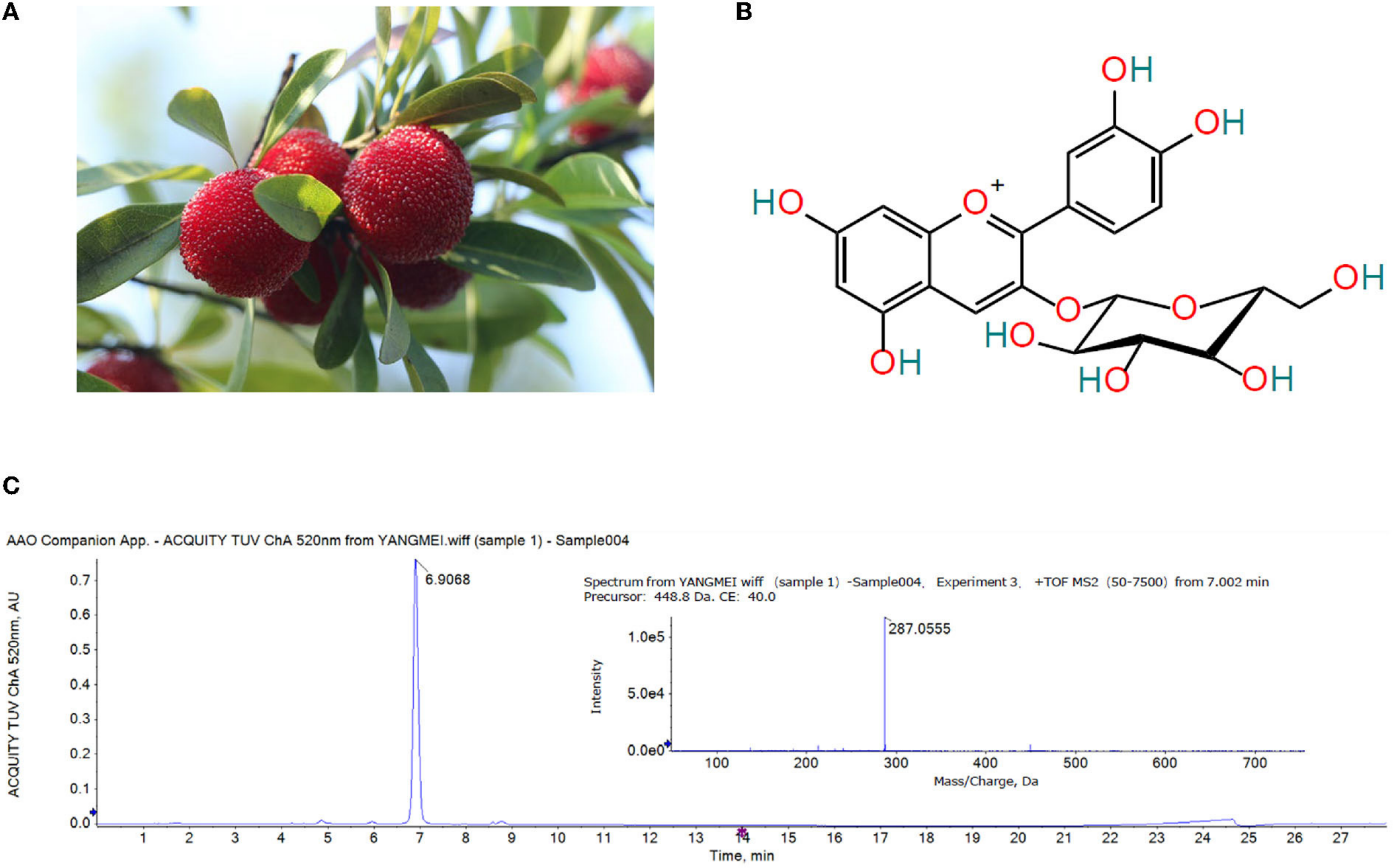
**(A)** Ripe ‘Dongkui' bayberry fruit **(B)** Structure of cyanidin-3-*O*-glucoside **(C)** UPLC chromatogram (λ = 520 nm) and MS/MS spectrum of the Chinese bayberry extract.

The Chinese bayberry variety used in this experiment is ‘Dongkui' (Figure 1A), which was harvested from Taizhou City, Zhejiang Province in June 2020. The bayberry fruits without mechanical damage, diseases and pests were selected and transported to the laboratory immediately after harvest and stored at 4°C for further experiments. Lincomycin Hydrochloride was purchased from Quanyu Biotechnology Animal Pharmaceutical Co., Ltd. (Shanghai, China). Montmorillonite powder was purchased from Zhejiang Hailisheng Pharmaceutical Co., Ltd. (Zhoushan, China). The ELISA kits of p-P65, TNF-α and IL-6, IL-12, IL-8, MIP-1α were purchased from Jiangsu Enzyme Immune Industrial Co., Ltd (Jiangsu, China). The first antibody against claudin-1 and ZO-1 was purchased from Santa Cruz Biotechnology, Inc. (claudin-1 item number is SC-81796, ZO-1 item number is SC-33725). High-throughput 16S rRNA gene sequencing of mouse feces was performed on an Illumina Miseq platform at Majorbio Bio-Pharm Technology Co., Ltd. (Shanghai, China). Other solvents and reagents were all analytical grade.

### Extraction and purification of C3G from Chinese bayberry

A total of 500 g Chinese bayberry flesh was accurately weighed and added into 2,000 mL absolute ethanol containing 0.1% formic acid (m:v = 1:4). The bayberry was fully ground with a blender. The mixture was subsequently ultrasonically extracted with a frequency of 35 kHz at low temperature for 1 h and then left overnight. The mixture was first filtered by 4 layers of gauze and then filtered by 3 layers of filter paper. The filtrate was vacuum dried at 30°C until the organic solvent was completely volatilized, and the residual was the crude extract of bayberry anthocyanin. The residual was then dissolved in 100 mL double distilled water. After the solution was filtered with 0.22 μm membranes, the crude extract was purified with Sep-Pak C18 cartridge columns (20 cc, 5 g sorbent, Waters Corp., Milford, MA, USA). Briefly, 2 bed volume (BV) of methanol and 2 BV of double distilled water was added to the column successively to condition the column. An appropriate amount of samples were loaded and 20 BV of distilled water was added to remove the organic acids and sugars. And finally, extraction rich in bayberry anthocyanins was obtained by eluting with 2 BV of absolute methanol. The collected eluent was vacuum dried at 30°C in the shade until the organic solvents were completely volatilized.

### Identification of C3G from Chinese bayberry

The powder rich in Chinese bayberry anthocyanin (1 mg) was dissolved in 1 mL HPLC-grade methanol with 0.1% formic acid to prepare a solution of 1 mg/mL. After filtering with the 0.22 μm membrane, an appropriate amount of solution was used for UPLC-Q-TOF-MS/MS identification. The optimized UPLC detection conditions are as follows: the detection was performed on an ACQUITY UPLC^®^ HSS T3 (1.8 μm, 2.1 × 150 mm) column. The mobile phase with a flow rate of 0.3 mL/min consisted of two solvents: water containing 1% formic acid (mobile phase A) and acetonitrile (mobile phase B). The gradient elution procedure was as follows: 0–5 min, 5–15% B; 5–12 min, 15–25% B; 12–20 min, 25–60% B; 20–23 min, 60–100% B; 23–24 min, 100–5% B; 24–28min, 5% B. The detection wavelength was 520 nm, the column temperature was 50°C, and the injection volume was 4 μL.

Mass spectrometry was carried out on the AB Triple TOF 5,600^+^ system (AB SCIEX, Framingham, USA) equipped with an ESI (electrospray ionization source) system. The experiment was operated on both positive and negative ion mode with source voltage as 5,500 V (positive mode) and −4,500 V (negative mode). The scanning range of m/z was set at 100–1,500. The pressure of Gas 1 (Air) and Gas 2 was set to 55 psi. The pressure of curtain gas (CUR) was set to 35 psi. The ion source temperature (TEM) was 600°C (positive mode) and 550°C (negative mode) respectively. The declustering potential (DP) was 100 V and the collision energy (CE) was 10 V. The exact mass calibration was performed automatically before analysis by the calibration delivery system.

### Animal experiment design

**Figure 2:**
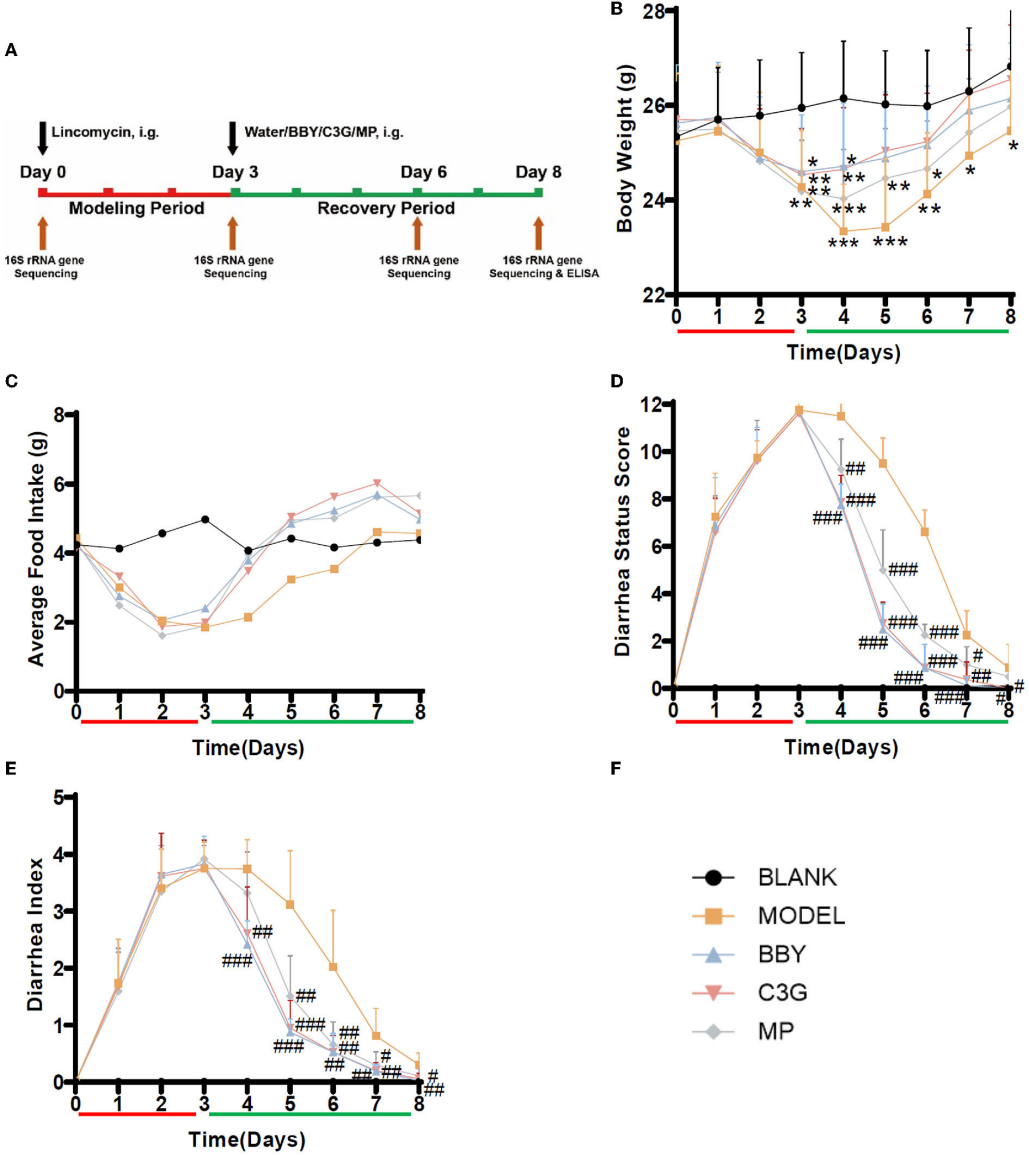
**(A)** Experimental scheme, black arrow indicates intragastric intervention, brown arrow indicates collection of fecal or tissue samples, red is the modeling stage, green is the recovery stage. **(B)** Average body weight change. **(C)** Average food intake. **(D)** Diarrhea status score, **(E)** Diarrhea index, *n* = 8/group, **(F)** Graph legends. The results were presented as mean ± standard deviation. *Indicated a significant difference compared with the BLANK group, # indicated a significant difference with the MODEL group, where *p < 0.05, **p < 0.01, ***p < 0.001; #*p* < 0.05, ##*p* < 0.01, ###*p* < 0.001.

The animal experiment was carried out with the approval of the Committee on the Ethics of Animal Experiments of Zhejiang University (permission number: ZJU20210210). Seven-week-old male BALB/c mice (22.3 ± 1.2 g) were purchased from Hangzhou Xuanzhu Biotechnology Co., Ltd., all mice were housed in a controlled environment with temperature 22 ± 0.5°C, humidity 50 ± 5% and a 12 h daylight cycle. After acclimation for a week, forty mice were randomly divided into 5 groups (*n* = 8 in each group): blank group (BLANK), antibiotic associated diarrhea model group (MODEL), group treated with bayberry dried powder (BBY), group treated with bayberry C3G (C3G) and a group treated with montmorillonite powder (MP). All mice had free access to food and water, and the drug was given by intragastric gavage. The experimental period was 8 days. The first 3 days were the modeling stage of AAD, and the last 5 days were the recovery stage of AAD (Figure 2A). During the modeling period, the diarrhea model was established by intragastric administration of lincomycin (3 g/kg) twice a day in MODEL, BBY, C3G and MP groups. In the recovery stage, mice in the BBY group were fed with dried bayberry powder suspension (100 mg/kg), C3G group were given C3G suspension (40 mg/kg), MP group were given montmorillonite powder suspension (40 mg/kg), mice in MODEL group were given the same amount of purified water, and the frequency and time of intragastric administration were the same as that of the model period for 5 days. The BLANK group did not receive any intervention. During the experiment, the changes in body weight and food intake of mice were recorded every day, and the diarrhea status score and diarrhea index were calculated by two persons according to the standard. The fecal samples of 5 mice in each group (fixed number) were taken on the 0, 3rd, 6th, and 8th day of the experiment, respectively. After snap-frozen with liquid nitrogen, they were stored at −80°C for further analysis. At the end of the experiment, that is, at the end of the 8th day, the mice were euthanatized to obtain colon specimens, some of which were soaked in formalin for histological evaluation and immunofluorescence, and the rest were stored at −80°C after snap-frozen for the detection of inflammatory factors.

### Evaluation of diarrhea

Diarrhea was evaluated according to diarrhea status score (Table 1) and diarrhea index (Table 2).

**Table 1:** Diarrhea score standard.

0	Normal mice
1	Loose stool, light color, perianal non-adhesive stool, general mental state
2	Anal adhesion stool, depression, loss of appetite, weight loss

**Table 2:** Criteria of diarrhea index.

**Sparse stool rate**	**Average sparse stool level** = **addition of sparse stool series/number of sparse stool**	
The number of diluted stools per animal/the total number of feces per animal	Grade 1	Stain diameter <1 cm
	Grade 2	Stain diameter 1–1.9 cm
	Grade 3	Stain diameter 2–3 cm
	Grade 4	Stain diameter >3 cm

Diarrhea index = Sparse stool rate × average dilute stool level.

### 16S rRNA gene sequencing

The following steps were conducted by Majorbio Bio-Pharm Technology Co., Ltd. (Shanghai, China). Raw fastq files were demultiplexed, quality-filtered by Trimmomatic and merged by FLASH based on the following criteria: (i) The reads were truncated at any site that received an average quality score <20 over a 50 bp sliding window. (ii) The primers were exactly matched, allowing a 2-nucleotide mismatch, and reads containing ambiguous bases were removed. (iii) Sequences with overlaps of longer than 10 bp were merged according to their overlap sequence. Operational taxonomic units (OTUs) were clustered with a 97% similarity cutoff using UPARSE (version7.1 http://drive5.com/uparse/), and chimeric sequences were identified and removed using UCHIME. The taxonomy of each 16S rRNA gene sequence was analyzed by the RDP Classifier algorithm (http://rdp.cme.msu.edu/) against the Silva 16S rRNA database (silva 132/16s bacteria) using a confidence threshold of 70%.

### Histological evaluation and immunofluorescence

Colon tissues were fixed in formalin and embedded in paraffin. Paraffin-embedded colon tissues were sectioned and stained with hematoxylin and eosin (HE). For immunofluorescence analysis, paraffin-embedded sections were deparaffinized with xylene and rehydrated with different proportions of ethanol. In order to prevent non-specific staining, the sections were incubated at room temperature in 4% bovine serum albumin (BSA) and 20% donkey serum phosphate buffer (PBS) for 1 h. The sections were incubated with anti-alkaline phosphatase (ALP) antibody (1:100) overnight at 4°C. After washing with PBS for 3 times (5 min each time), the sections were incubated with secondary antibody immunofluorescence for 2 h in the dark and then washed for 3 times in the dark. The immunostained sections were imaged with Olympus Fv3000.

### Measurement of inflammatory factors

The temperature of the colon tissue was kept at 2 ~8°C after thawing. Colon tissues were fully homogenized in PBS (pH = 7.4) with a homogenizer. The supernatant was collected carefully after centrifuging at 3,000 rpm for 20 min. The levels of p65 phosphorylation, TNF-α, IL-6, IL-12, IL-8 and MIP-1α were determined by ELISA kits according to the manufacturers' instructions.

### Statistical analysis

Statistical analysis and visualization of the results were performed with GraphPad Prism software 8 (GraphPad Software, San Diego, CA, USA), Excel 2016 and SPSS software (SPSS 23.0 for Windows, IBM, Chicago, IL, USA). Independent Samples Student's *t*-test was used for two groups comparison. Except for special explanation, *p* < 0.05 was considered statistically significant.

## Results

### Analysis of anthocyanins in Chinese bayberry

The anthocyanins in the extract of Chinese bayberry were analyzed by UPLC-Q-TOF-MS/MS. As showed in Figure 1C, the MS/MS fragment ion at m/z 287.0555 was (C_21_H_20_O_11_-glucoside + H)^+^, the precursor of cyanidin, indicative of cyanidin-glycoside. The base MS peak at m/z 449.1088 was (C_21_H_20_O_11_)^+^, so it was inferred that the compound was cyanidin-3-*O*-glucoside (Figure 1B).

### Therapeutic effects of Chinese bayberry and C3G-rich extract on AAD in mice

After the modeling period (Figure 2A), the mice treated with lincomycin showed symptoms of diarrhea, that is, slow movement, decreased food intake and body weight, and the diarrhea rate was 100%, suggesting that the establishment of AAD mice model was successful. During the modeling period, the bodyweight of the model mice decreased, the extent of the decrease was consistent, and there was no difference between groups. On the first day of recovery, the bodyweight of mice in BBY and C3G groups began to increase and no longer decreased, while the bodyweight of mice in MODEL and MP groups still decreased (Figure 2B). After Day 5, there was no significant difference in body weight between BBY, C3G groups and BLANK group, however, the bodyweight of MODEL group kept significantly lower than that of BLANK group to the end of the experiment. The average daily food intake (Figure 2C), diarrhea status score (Figure 2D) and diarrhea index (Figure 2E) reflected the same trend. The changes of perianal condition and stool condition of mice can be seen intuitively in (Supplementary Figures 1, 2). Therefore, compared with the natural recovery MODEL group, the treatment with dried bayberry powder, C3G and montmorillonite powder could rapidly reduce the diarrhea score and diarrhea index (Figure 2B), increase food intake and restore body weight gain, and the therapeutic effect was ranked as BBY ≈ C3G > MP > MODEL.

### Gut microbiota regulatory effect of Chinese bayberry and C3G-rich extract on AAD in mice

**Figure 3:**
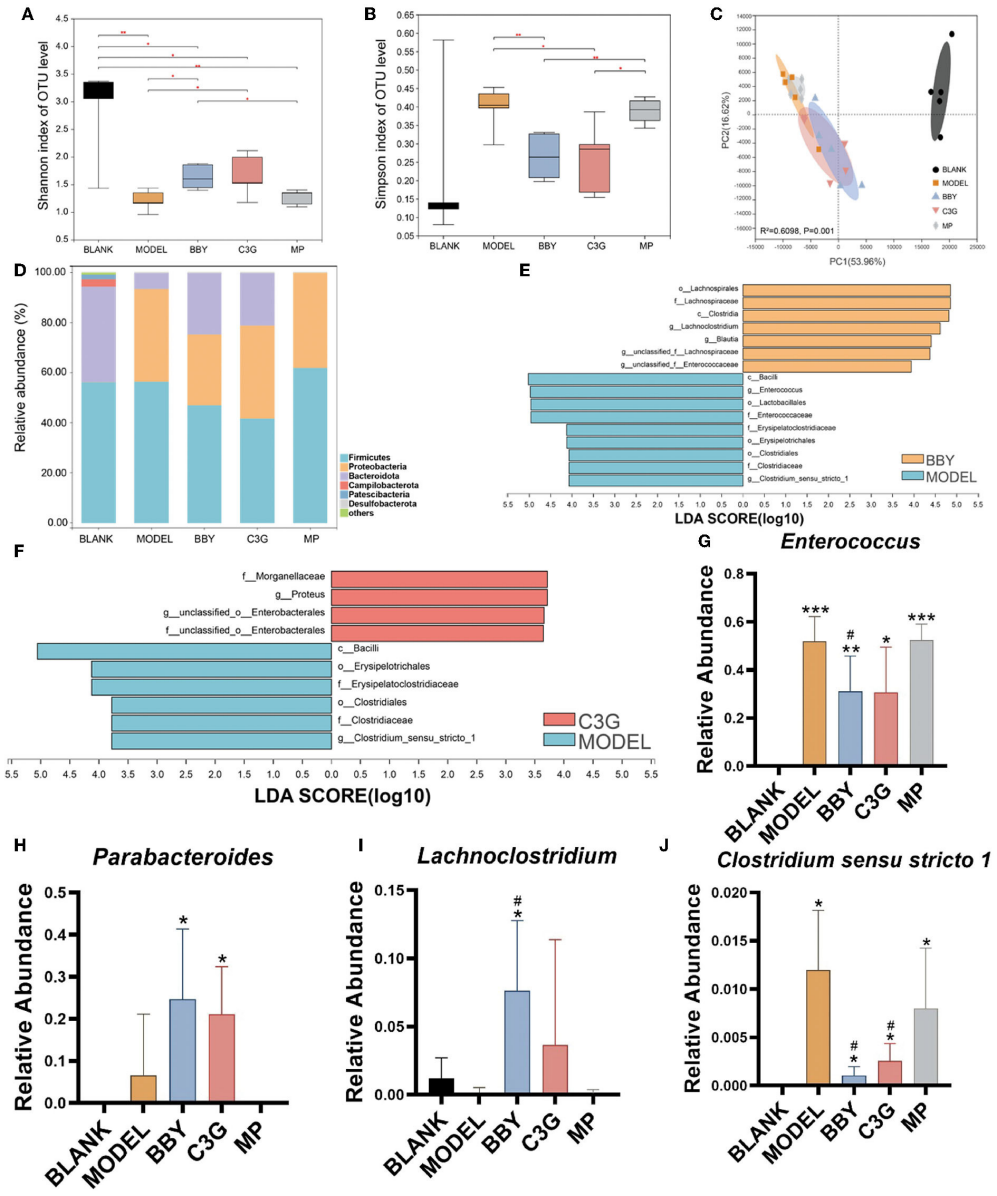
**(A)** Shannon index. **(B)** Simpson index. **(C)** PCA on Family level. **(D)** The composition of gut microbiota in each group at phylum level. **(E,F)** LEfSe analysis of BBY-MODEL group and C3G-MODEL group. **(G–J)** Relative abundance of bacteria with significant difference at genus level. The results were expressed as mean ± standard deviation. *Indicated a significant difference compared with the BLANK group, # indicated a significant difference with the MODEL group, where *p < 0.05, **p < 0.01, ***p < 0.001; #*p* < 0.05, (*n* = 5/group).

Simpson and Shannon index (Figures 3A,B) represented the richness and diversity of gut microbiota. The results showed that the richness and diversity of mice gut microbiota after modeling were significantly lower than those of BLANK group. However, compared with MODEL group, the richness and diversity of BBY and C3G treatments were significantly increased (*p* < 0.05), and there was no significant difference between BBY and C3G groups. The recovery was not significant in MP group. Principal component analysis (PCA) (Figure 3C) indicated that, compared with the BLANK group, the gut microbiota composition of MODEL, BBY, C3G and MP groups changed significantly along the PC1 direction. On the other hand, separation occurred in MODEL, MP, BBY and C3G groups along PC2. As displayed in Figure 3C, MODEL and MP groups were clustered closely, which significantly distinguished with the cluster composed by BBY and C3G groups. Compared with MODEL and MP groups, the proportion of Bacteroides in BBY and C3G groups increased at phylum level (Figure 3D). In order to find out the specific bacteria that led to the alteration of the gut microbiota, we further performed linear discriminant analysis (LDA) coupled with effect size measurements (LEfSe) to get dominant bacteria between MODEL, BBY and C3G groups, and the results were shown in Figures 3E,F. After the predominant bacteria were identified, we next performed difference analysis at genus level. As displayed in Figures 3G–J, the relative abundance of *Enterococcus* and *Clostridium senus stricto 1* in BBY and C3G groups decreased significantly, while the relative abundance of *Parabacteroides* and *Lachnoclostridium* enriched significantly compared with MODEL group. *Enterococcus* and *Clostridium senus stricto 1* are more likely to be considered as potential pathogens, while *Parabacteroides* and *Lachnoclostridium* are more likely to be beneficial bacteria, which are thought to be related to anti-obesity effect, etc. More details, such as rarefaction curves and hierarchical clustering tree, can be seen in the (Supplementary Figures 3–7).

**Figure 4:**
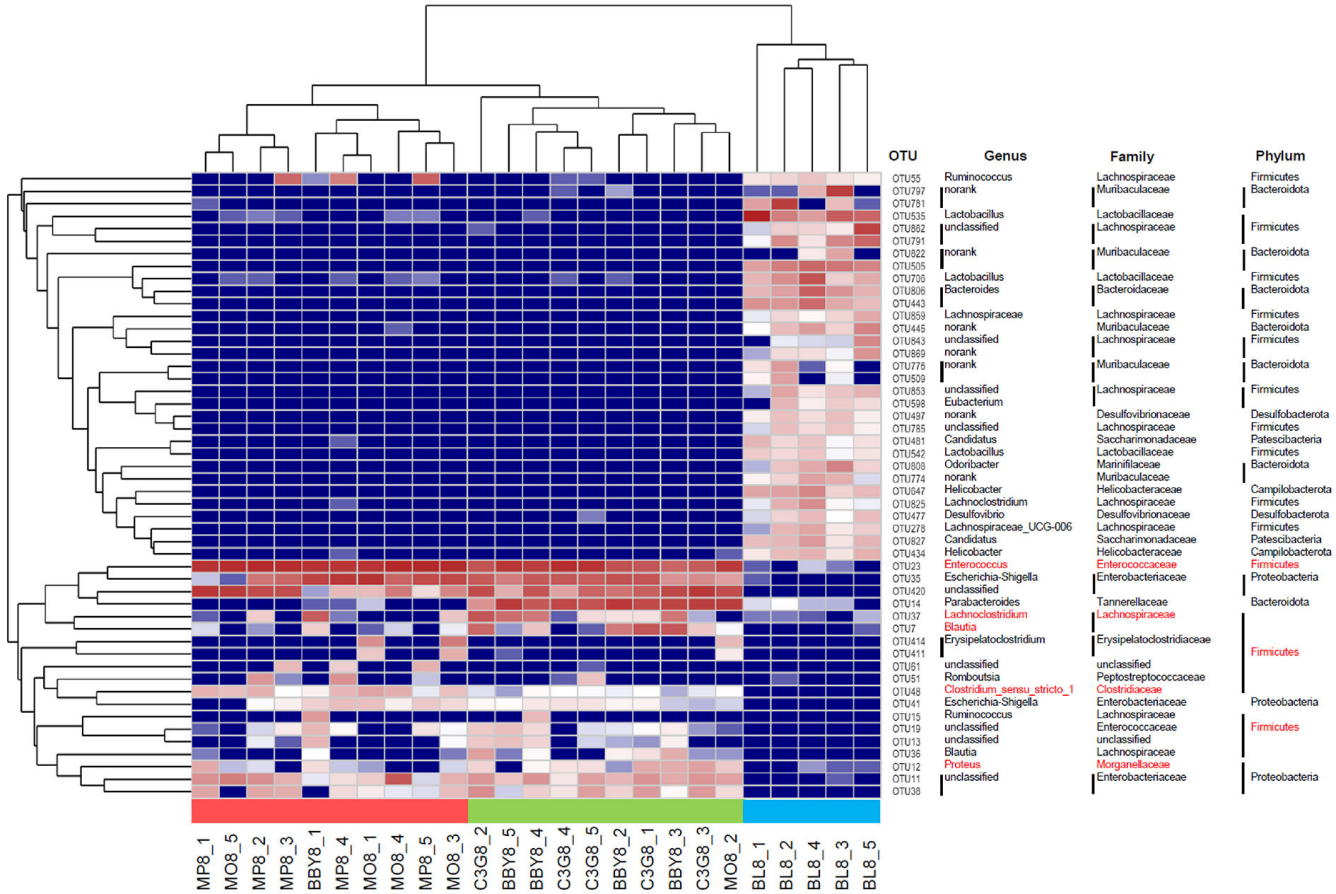
Heatmap based on the relative abundance of bacteria in each sample at OTU level. Species labeled red were bacteria that most likely to explain differences between groups reflected by LEfSe analysis.

The sequencing results of 25 fecal samples collected at the end of the experiment were clustered at OTU level, and the results were shown in Figure 4. Similar to the result of PCA, samples of MODEL group and MP group were nearly clustered together, while BBY group and C3G group were almost clustered together, and BLANK group was clustered separately. The bacteria that might be of biological significance were labeled red and classified at phylum, family and genus levels.

### Chinese bayberry powder and C3G up-regulated the expression of intestinal tight junction protein

**Figure 5:**
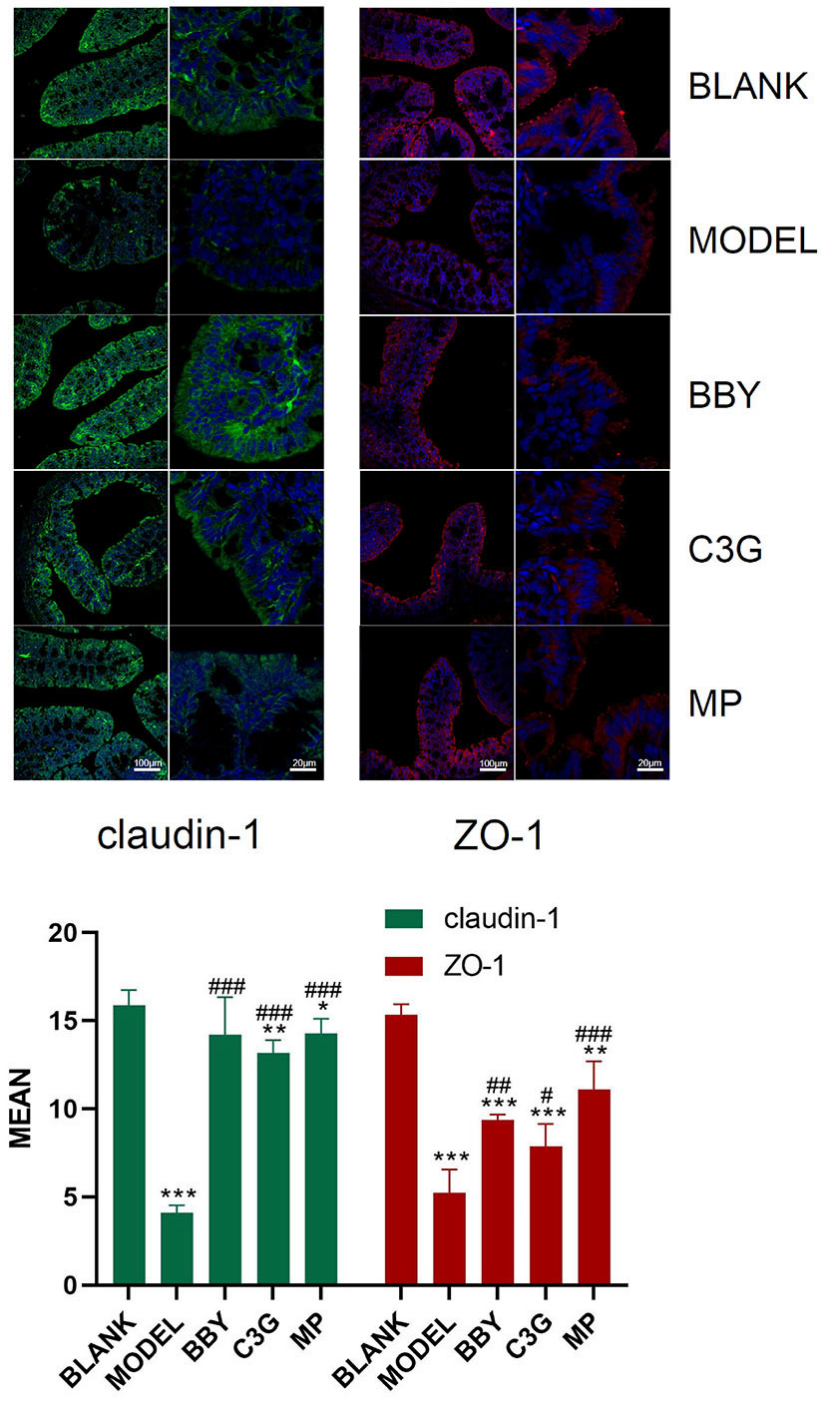
Representative images of immunofluorescence of claudin-1 and ZO-1. Green fluorescence is claudin-1, red fluorescence is ZO-1, and blue fluorescence is DAPI staining. The figure below is a quantitative comparison of the fluorescence intensity calculated by ImageJ. *Indicated a significant difference compared with the BLANK group, # indicated a significant difference with the MODEL group, where *p < 0.05, **p < 0.01, ***p < 0.001; #*p* < 0.05, ##*p* < 0.01, ###*p* < 0.001.

The colonic epithelial cells of BLANK mice showed persistent and strong immunofluorescence of claudin-1 and ZO-1, while those of MODEL mice showed weak and discontinuous immunofluorescence. The brightness and continuity of immunofluorescence of BBY, C3G and MP groups were between BLANK and MODEL groups (Figure 5). The results showed that intestinal tight junction protein was greatly damaged and the intestinal barrier was impaired in mice with antibiotic diarrhea, while intake of dried bayberry powder and anthocyanin could up-regulate the expression of intestinal tight junction protein and restore the integrity of the intestinal barrier.

### C3G alleviated inflammation through NF-κB signal pathway

**Figure 6:**
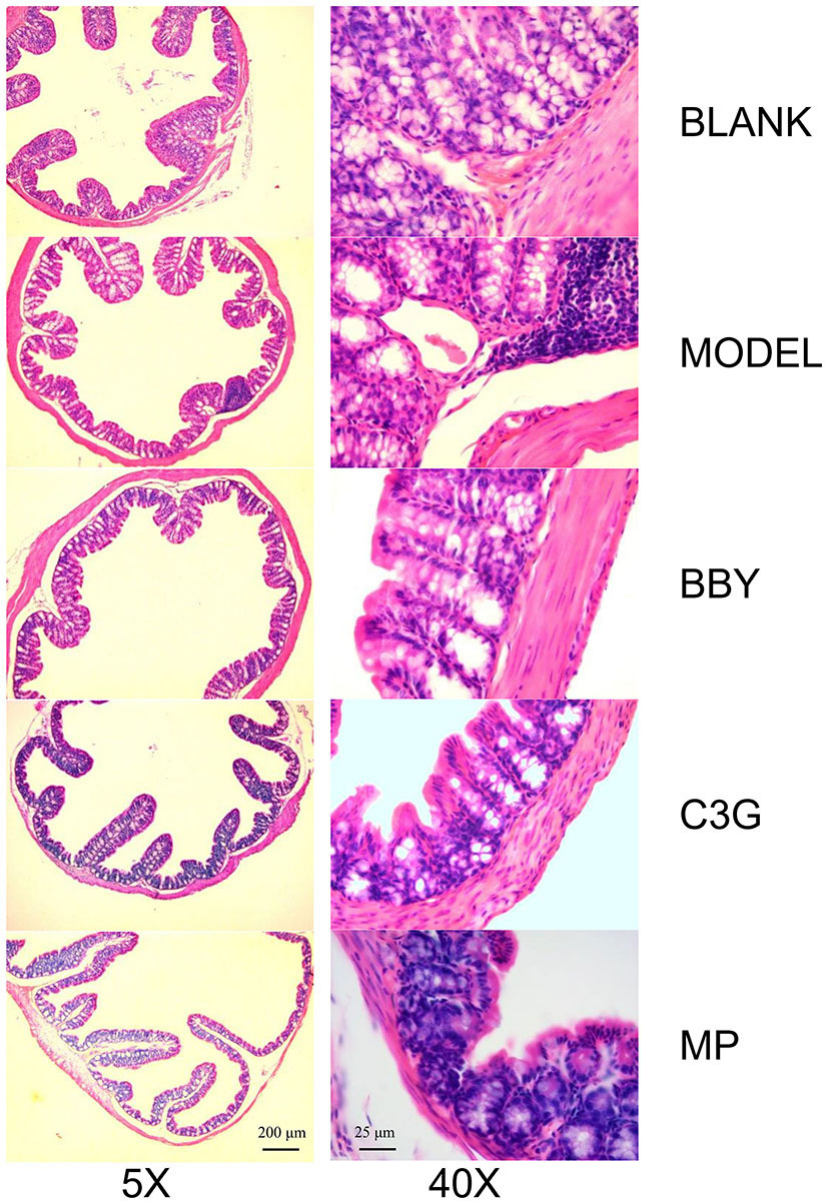
Representative images of hematoxylin and eosin-stained colonic sections under 5X (left) and 40X multiples.

Through the observation of HE staining pictures of mice colon (Figure 6), it was found that compared with BLANK group, the intestinal structure of each group was complete, and the morphology and spacing of intestinal recess were basically normal. However, a large number of lymphocyte infiltration could be seen in the lamina propria of MODEL group and MP group, but rarely in BBY group and C3G group.

**Figure 7:**
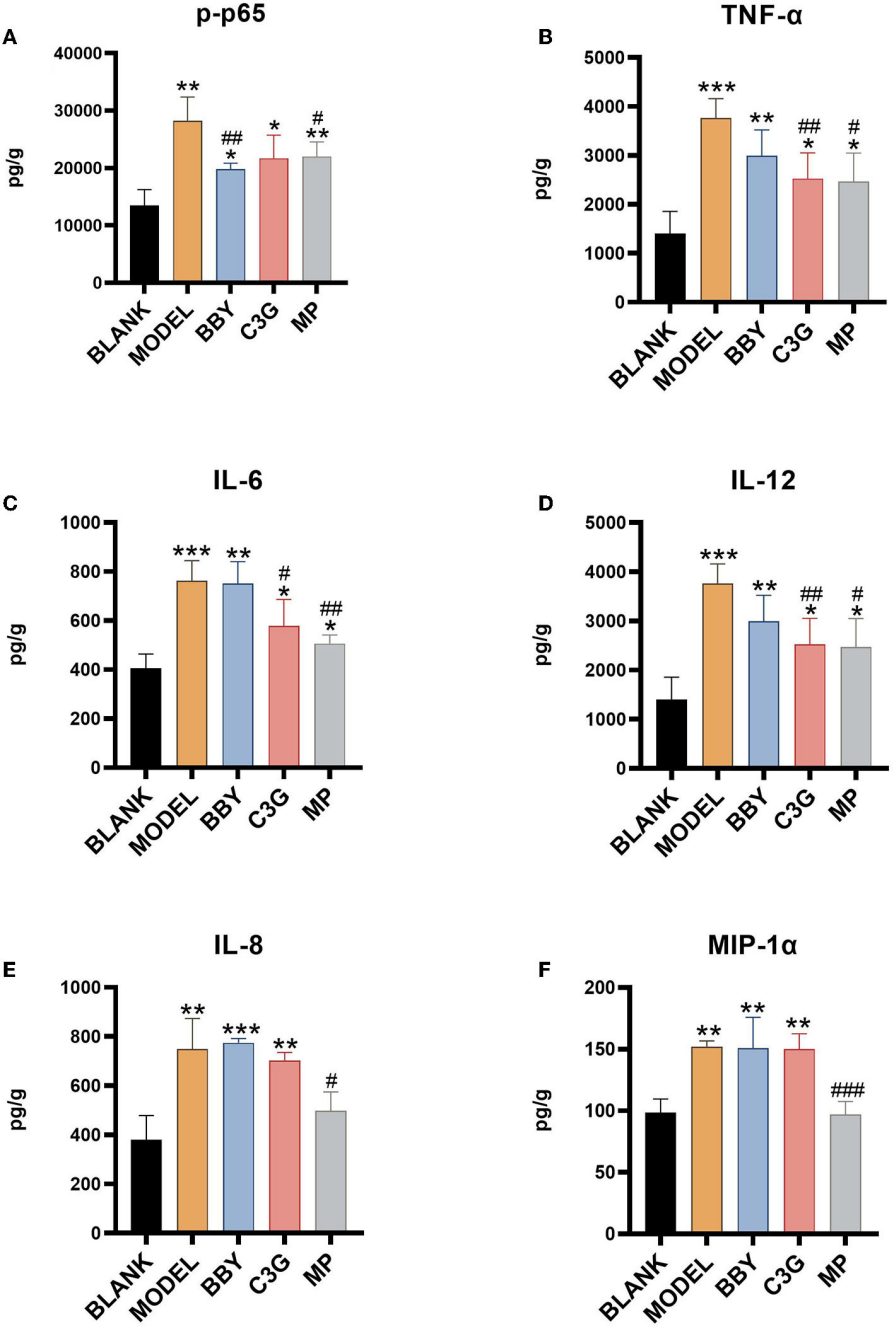
**(A)** p65 phosphorylation level, **(B)** TNF-α level, **(C)** IL-6 level, **(D)** IL-12 level, **(E)** IL-8 level, **(F)** MIP-1α level. *Indicated a significant difference compared with the BLANK group, # indicated a significant difference with the MODEL group, where *p < 0.05, **p < 0.01, ***p < 0.001; #*p* < 0.05, ##*p* < 0.01, ###*p* < 0.001, (*n*= 5/group).

The levels of inflammatory factors of MODEL group were significantly higher than those of BLANK group. As shown in Figure 7, intake of Chinese bayberry powder and C3G significantly decreased the level of p65 phosphorylation. C3G treatment also significantly decreased the level of TNF-α, IL-6 and IL-12, BBY group showed a similar trend, but the difference was not significant. TNF-α and IL-6, IL-12 belong to the inflammatory facilitation pathway in the downstream pathway of NF-κB, and IL-8, MIP-1α belongs to the inflammatory chemotaxis pathway in the downstream pathway of NF-κB. Therefore, red Chinese bayberry pulp components, especially C3G, could alleviate the inflammatory response of antibiotic-associated diarrhea by down-regulating the level of IL-6, IL-12 factors and inhibiting the inflammatory facilitation of NF-κB pathway (Figure 7).

## Discussion

Chinese bayberry is a subtropical fruit native to China, which has been cultivated in China for more than 2,000 years. Chinese bayberry received great popularity because of its delicious taste, attractive color and high health value (10). In traditional Chinese medicine, Chinese bayberry soaked in distilled wine is often used to treat diarrhea (9). In recent years, the mechanism of the therapeutic effect of Chinese bayberry fruit extract on diarrhea has been partially revealed. Studies have proved that Chinese bayberry was able to alleviate food poisoning diarrhea through the inhibition of salmonella, however, the efficacy on AAD with different pathogenesis has never been systematically and scientifically demonstrated (11). AAD is a major complication of antibiotic therapy for diarrhea, which is related to gut microbiota dysbiosis, inflammation and mucosal injury (6, 7). In order to test our hypothesis that Chinese bayberry could exert therapeutic effect on AAD through regulation of gut microbiota, we established a mice AAD model by lincomycin. Then the successfully established AAD model mice were intragastrically fed with Chinese bayberry powder, C3G and montmorillonite powder, respectively. Our results revealed that red bayberry fruit had obvious anti-diarrhea effect, mainly reflected by quickly reversing the trend of weight loss, decreasing diarrhea status score and diarrhea index. We confirmed for the first time that Chinese bayberry fruit had a good therapeutic effect on AAD, and in subsequent experiments, we found that the effect was realized through positively regulating gut microbiota, reducing inflammation and restoring intestinal mucosal barrier. And we surprisingly found that the effect was as good as equivalent dose of montmorillonite powder.

**Figure 8:**
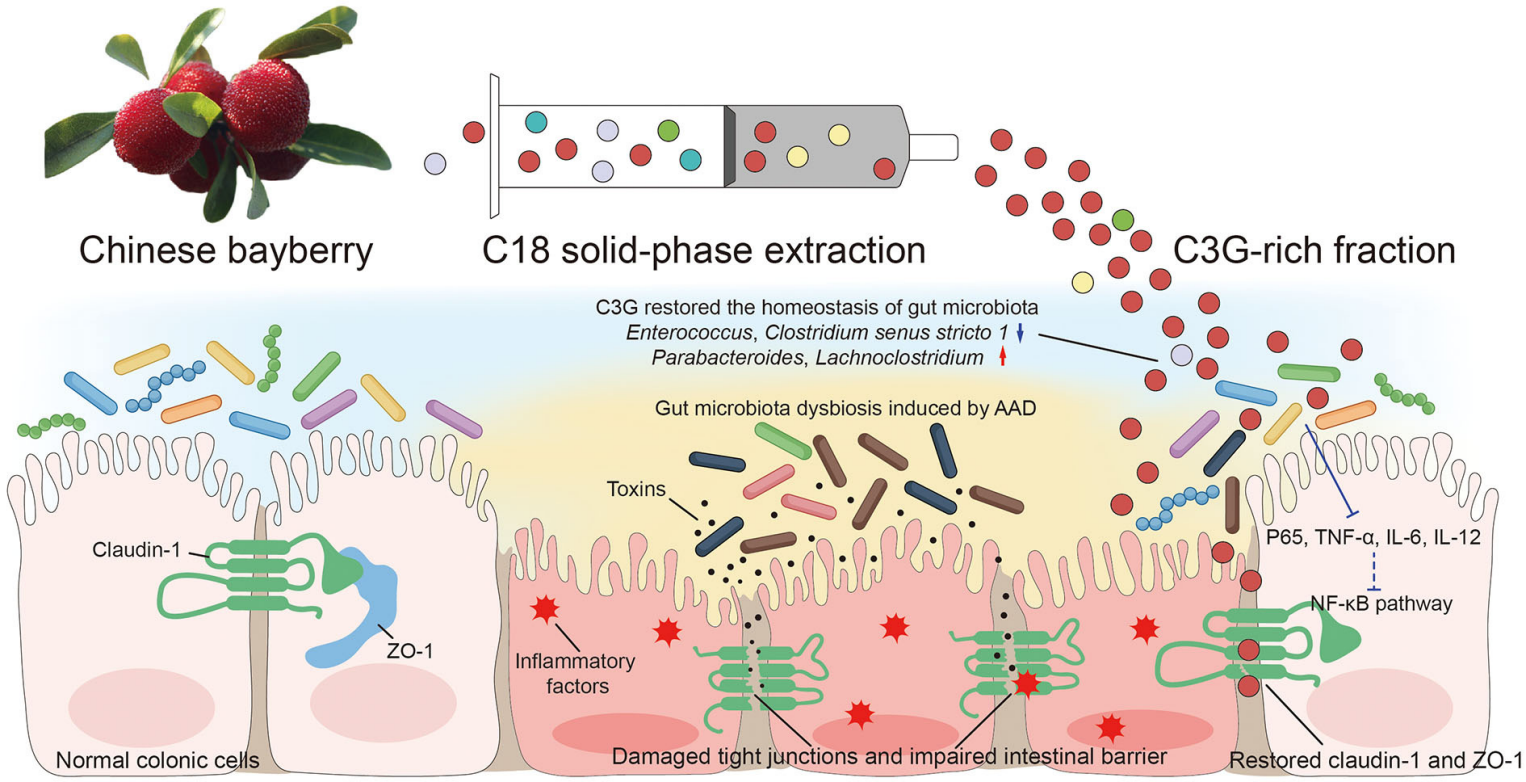
Schematic diagram showing the possible mechanisms of alleviating AAD effects of Chinese bayberry.

C3G is a kind of flavonoid and is the most widely distributed water-soluble pigment in nature, which widely exists in human diet. In the past few decades, the effects of C3G on health promotion and disease prevention have been widely studied (18–20). Studies have shown that dietary polyphenols can regulate intestinal inflammation and inhibit the pathogenesis of inflammatory bowel disease (IBD) (21). IBD is a chronic, recurrent gastrointestinal inflammatory disease, including Crohn's disease (CD) and ulcerative colitis (UC) (22). C3G mainly inhibits the activation of inflammatory pathways regulated by NF-κB. Typical NF-κB is a heterodimer composed of p50 and p65 subunits. In unstimulated cells, NF-κB dimer is isolated in the cytoplasm by IκBs (κB inhibitor). Cells under various stimuli will trigger cascade signal response, p65 is phosphorylated, resulting in IκBs destruction and nuclear translocation of NF-κB, activation of a series of pro-inflammatory genes, resulting in increased levels of pro-inflammatory cytokines TNF-α, IL-6, and IL-12 (23, 24). These factors are the mediators of immune response and contribute to the activation and expansion of Th1 cell-mediated inflammatory response pathway. C3G could prevent p65 nuclear translocation in a dose-dependent manner and restore it to a level similar to that of unstimulated cells, thus inhibiting the activation of inflammatory pathways. The intestinal inflammation in AAD was far less drastic than that in IBD. The levels of inflammatory cytokines TNF-α, IL-6, and IL-12 in colonic tissue of mice fed with C3G were significantly lower than those of MODEL group, which was highly consistent with the result of p-p65. It is worth mentioning that the level of IL-8 and MIP-1α which are the inflammatory chemokines of downstream NF-κB pathway, were not lower than that of MODEL group. In view of the complexity of the components of bayberry fruit, C3G extracted from bayberry fruit with a purity of 95% showed a better effect on decreasing inflammatory factors than that of bayberry powder. We supposed that C3G in Chinese bayberry played the main role in the therapy of AAD and intestinal inflammation. In order to express our research more intuitively, we made the schematic diagram (Figure 8).

Gut microbiota is a complex ecosystem, and there are great interactions between microorganisms and the hosts (4). Although antibiotics target specific types of microorganisms, their effects on microbial ecology are not limited to target microbes. For example, killing or inhibiting some kinds of bacteria favors the growth of other kinds of microorganisms, which in turn leads to microbial community dysbiosis, making it possible for the proliferation of some potential pathogenic bacteria (25). Antibiotics are aimed at specific pathogenic microorganisms, but their effects on gut microbiota are profound, enduring and unpredictable. Epidemiologically, it's often associated with a variety of long-term health problems, including obesity, asthma, allergy and IBD (26). AAD is one of the acute manifestations of gut microbiota dysbiosis. Restoring the homeostasis of gut microbiota has become the key to the treat AAD. Compared with BLANK group, the richness and diversity of gut microbiota decreased in all mice fed with lincomycin, and the species of bacteria in intestinal contents decreased in different degrees at the level of phylum, class, order, family and genus, which indicated that antibiotics destroyed the homeostasis of normal gut microbiota, resulting in gut microbiota disorder, and the F/B ratio of AAD mice increased significantly, which was consistent with previous research results. After 5 days of treatment, the richness and diversity of gut microbiota in BBY and C3G groups were significantly increased compared with those in MODEL group, and the ratio of F/B was also decreased, but there was no difference between MP group and MODEL group. The proportion of microbiota at the phylum level showed that the composition of BBY and C3G group was similar, while the composition of MODEL group was consistent with that of MP group, and the proportion of Bacteroides was significantly increased in BBY and C3G group. Bacteroides are strictly anaerobic gram-negative bacteria, which are the dominant bacteria in the human intestinal tract, accounting for nearly 25% of the total gut microbiota (27). Their roles in the intestinal tract include regulating intestinal microenvironment, such as redox levels, participating in carbohydrate metabolism, regulating immune response, secreting short-chain fatty acids (SCFAs) such as acetic acid, propionic acid and butyric acid. Butyrate plays a key role in improving intestinal barrier function (28). At genus level, the relative abundance of *Enterococcus* and *Clostridium senus stricto 1* in BBY and C3G groups were significantly lower than those in MODEL group. *Enterococcus* represented by *E. faecalis* and *E. faecium*, is an important opportunistic pathogen. *Enterococcus* can produce a variety of toxic factors, which help adhesion, colonization and invasion of host tissue, regulation of host immunity and production of extracellular enzymes and toxins (29, 30). *Clostridium senus stricto 1* has been found to proliferate in a variety of intestinal inflammatory diseases such as IBD and IBS, and can be used as a biomarker of intestinal inflammation (31). The relative abundance of *Lachnoclostridium* in BBY and C3G groups increased compared with MODEL group. Diarrhea will lead to a decrease in the concentration of butyric acid in the intestine of mice, and *Lachnospiraceae* has been shown to produce both butyrate and propionate, which helps to restore the concentration of SCFAs in the intestine (32). SCFAs, as the ligand of two G protein coupled receptors (GPCRs) Gpr41 and Gpr43, participate in glycolysis and protein synthesis by regulating the level of many endocrine peptides, promote the proliferation, differentiation and apoptosis of intestinal epithelial cells, and help to protect the intestinal epithelial barrier (33, 34). In addition, *Parabacteroides* (35) and *Blautia* (36, 37) are more considered to be beneficial bacteria and have been found to play a positive role in anti-obesity, anti-intestinal inflammation and protection of intestinal epithelial barrier.

Intestinal mucosal barrier dysfunction plays an important role in the occurrence and development of intestinal inflammation (38). As a key component of the intestinal mucosal barrier, tight junction proteins close the gap between adjacent intestinal epithelial cells and retain antigens and microorganisms in the intestinal lumen, which play an important role in maintaining intestinal permeability, tissue differentiation and internal environment stability (37). In our experiment, intestinal tight junction protein was impaired and the intestinal barrier was damaged in MODEL group, while BBY and C3G group could up-regulate the expression of intestinal tight junction protein and restore the integrity of the intestinal barrier. We summarized that C3G restored the intestinal barrier through direct and indirect ways. The direct effects of C3G were as follows: first, protocatechuic acid (PCA), the metabolite of C3G, could reduce intestinal permeability and repair intestinal barrier function by regulating the expression level of ZO-1 tight junction protein; second, C3G could maintain the integrity of intestinal barrier by regulating the response of mucosal immune cells. TNF-α could enlarge local or systemic inflammation, causing disorders of tight junction protein and intestinal mucosal barrier function. The indirect effects of C3G were as follows: first, C3G maintained intestinal mucosal barrier function by reducing the expression of inflammatory factors such as TNF-α and promoting the expression of tight junction proteins such as claudin-1 and ZO-1; second, probiotics such as *Lachnoclostridium* and *Blautia* can inhibit pathogens and enhance the expression of tight junction protein in intestinal epithelial cells by producing antibacterial substances such as lactic acid and SCFAs and competing for nutrients and intestinal adhesion sites, thus reducing intestinal permeability (32, 37). In addition, the latest research showed that montmorillonite powder mainly ameliorated antibiotic diarrhea by protecting intestinal mucosa, reducing intestinal permeability and thus reducing inflammation, and had little effect on gut microbiota (39). Therefore, montmorillonite powder can be an excellent one-way positive control drug for in-depth study of the proportion of gut microbiota disorder and increased intestinal permeability in diarrhea. Of course, it is not limited to diarrhea, but also in other hot areas of gut microbiota.

Chinese bayberry fruit had the effect of alleviating AAD, and C3G was supposed to play the main role. Its mechanism was to restore the homeostasis of gut microbiota, inhibit the level of harmful bacteria, increase the abundance of beneficial bacteria, down-regulate TNF-α, IL-6, and IL-12 factors to reduce inflammation, restore intestinal tight junction proteins and reduce intestinal permeability. Our findings provided novel insights into the therapeutic effect of C3G on AAD, and suggested that the Chinese bayberry could be a promising functional food on AAD treatment.

## Data availability statement

The raw data supporting the conclusions of this article will be made available by the authors, without undue reservation.

## Ethics statement

The animal study was reviewed and approved by the Committee on the Ethics of Animal Experiments of Zhejiang University (permission number: ZJU20210210).

## Author contributions

YSW: conceptualization, investigation, formal analysis, and writing—original draft. JBC: conceptualization, methodology, and writing—review and editing. YW: conceptualization, investigation, and writing—review and editing. FHZ: validation, data curation, and methodology. MYQ: investigation, methodology, and funding acquisition. ZWH and JLY: investigation and data curation. FPB: investigation and funding acquisition. XL, CDS, and YXZ: supervision, conceptualization, and funding acquisition. All authors contributed to the article and approved the submitted version.

## Funding

The authors would like to acknowledge the financial support provided by the National Natural Science Foundation of China (32101932), the Key Research and Development Program of Zhejiang Province (2021C02018 and 2020C01022), and the Experimental Animal of Public Welfare Research Project of Zhejiang Province (LGD20H090006).

## Conflict of interest

The authors declare that the research was conducted in the absence of any commercial or financial relationships that could be construed as a potential conflict of interest.

## Publisher's note

All claims expressed in this article are solely those of the authors and do not necessarily represent those of their affiliated organizations, or those of the publisher, the editors and the reviewers. Any product that may be evaluated in this article, or claim that may be made by its manufacturer, is not guaranteed or endorsed by the publisher.
