# Supplementary material for: Cyanidin-3-O-glucoside extracted from the Chinese bayberry (Myrica rubra Sieb. et Zucc.) alleviates antibiotic-associated diarrhea by regulating gut microbiota and down-regulating inflammatory factors in NF-κB pathway
Source: Front Nutr. 2022 Aug 24;9:970530. doi: 10.3389/fnut.2022.970530 (PMC9449314; doi:10.3389/fnut.2022.970530)
Supplement: Supplementary file 1 [file Data_Sheet_1.PDF]

Supplementary Material

1 Supplementary Figures 1

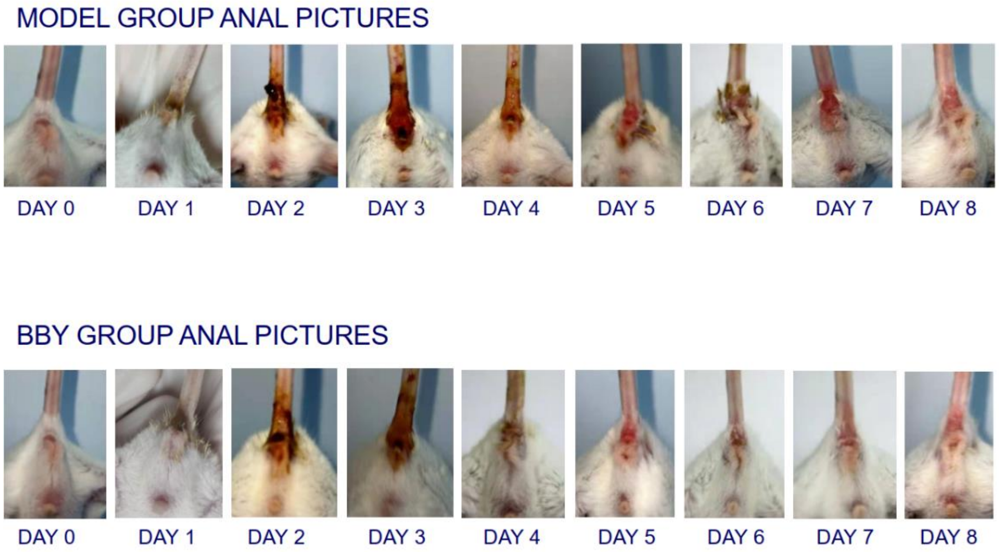

Supplementary Figure 1. Perianal pictures taken every day in MODEL group and BBY group during the experiment.

2 Supplementary Figures 2

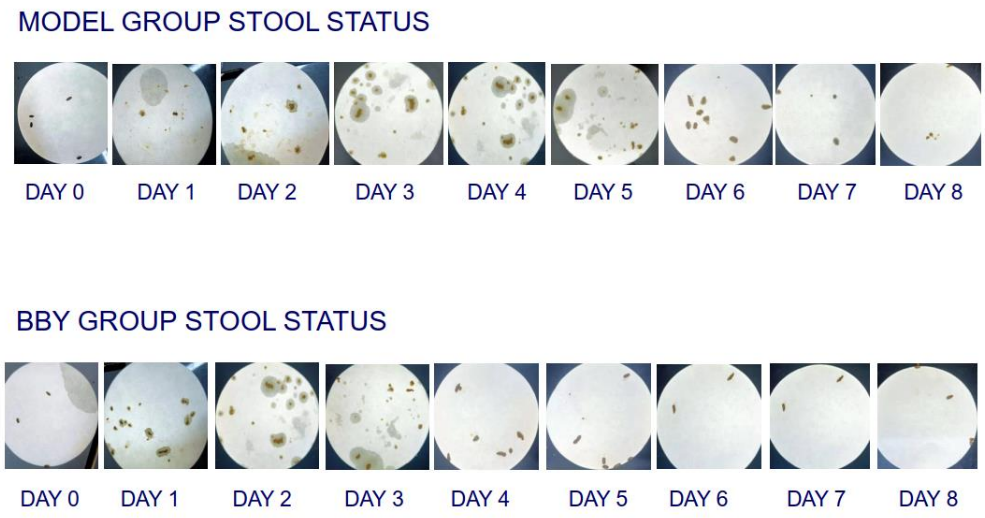

Supplementary Figure 2. Stool status pictures taken every day in MODEL group and BBY group during the experiment.

3 Supplementary Figures 3

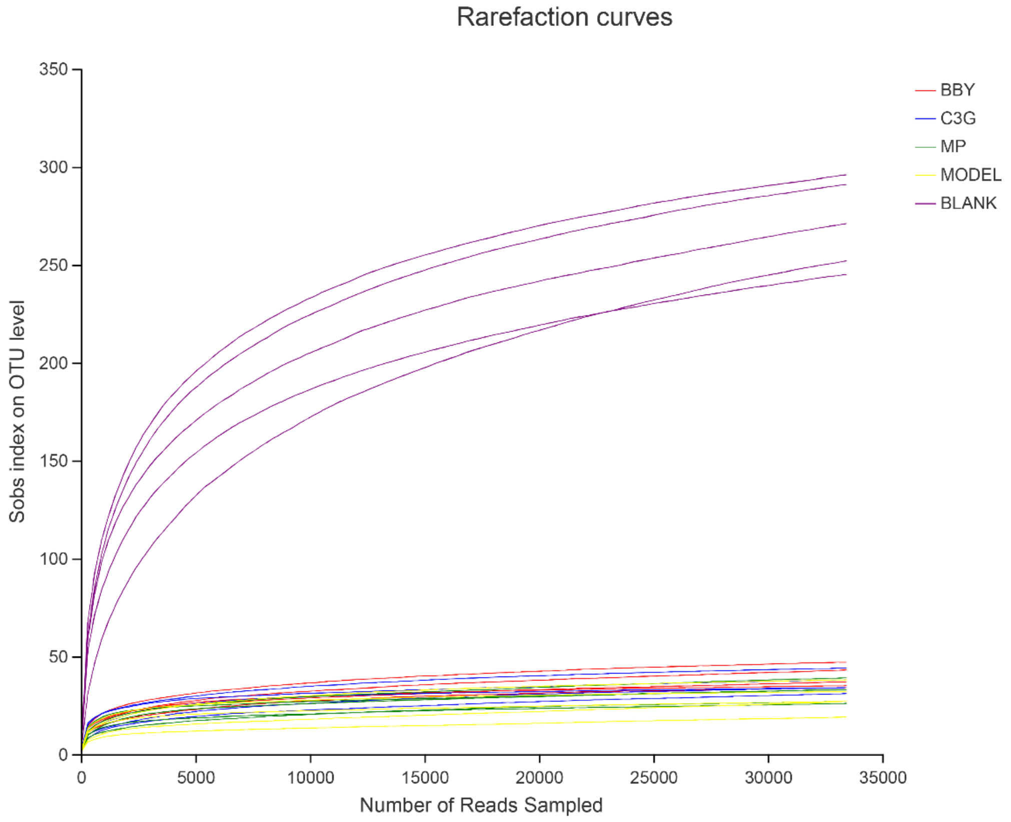

Supplementary Figure 3. Rarefaction curves of all stool samples on OTU level..

4 Supplementary Figures 4

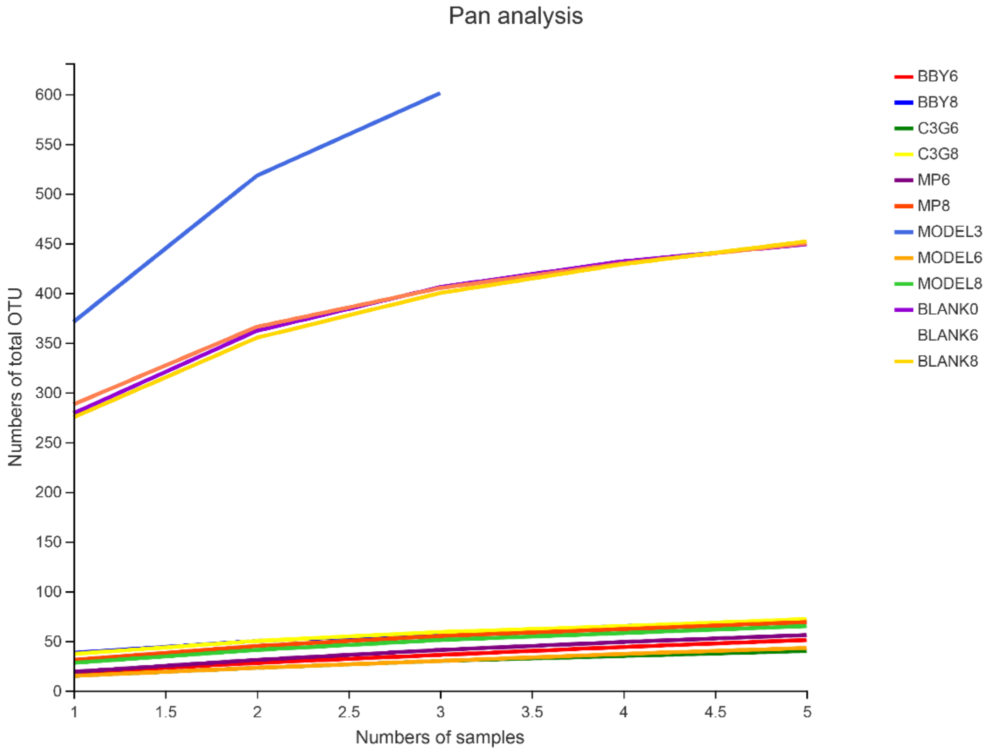

Supplementary Figure 4. Pan analysis of all stool samples on OTU level..

5 Supplementary Figures 5

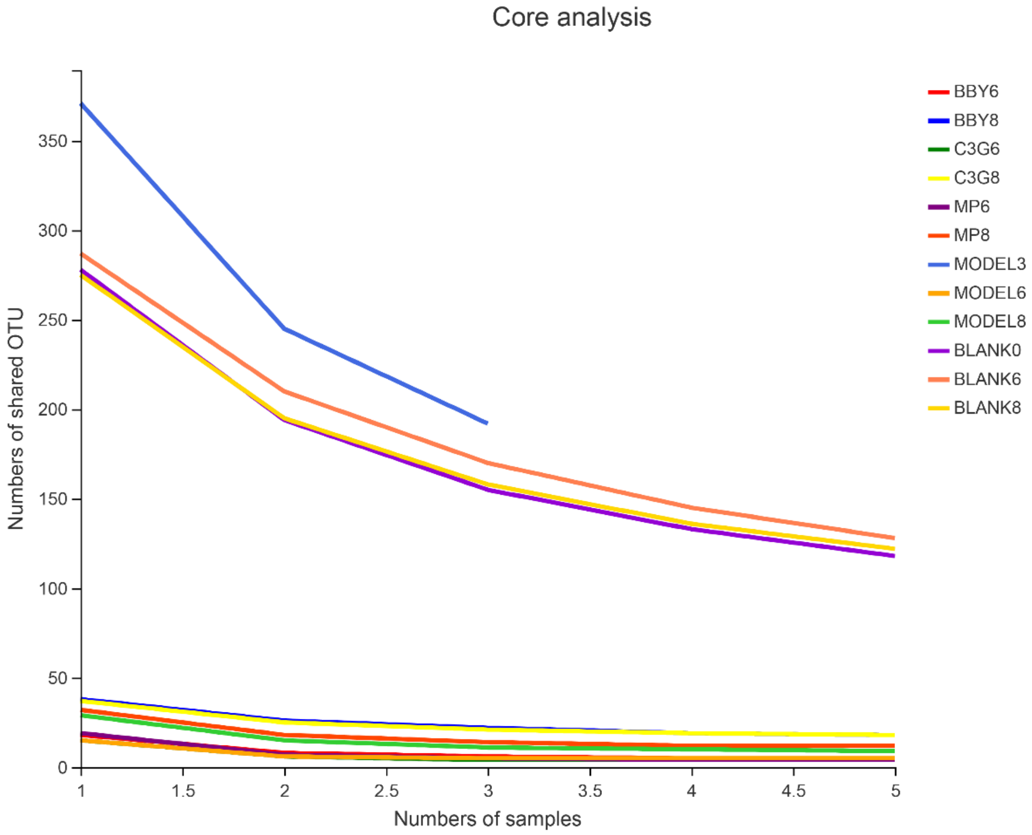

Supplementary Figure 5. Core analysis of all stool samples on OTU level.

6 Supplementary Figures 6

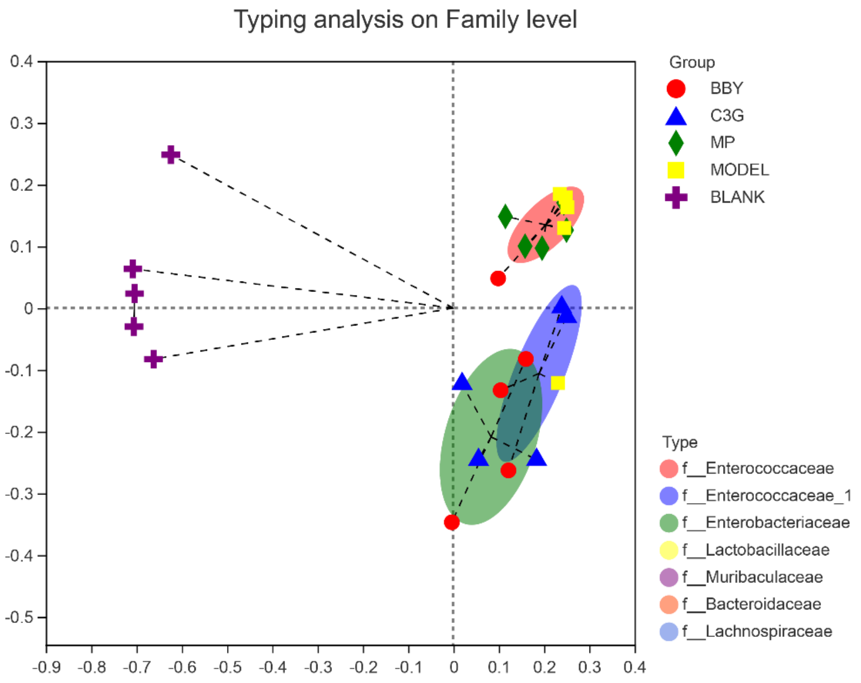

Supplementary Figure 6. Typing analysis on Family level of stool samples in day 8.

7 Supplementary Figures 7

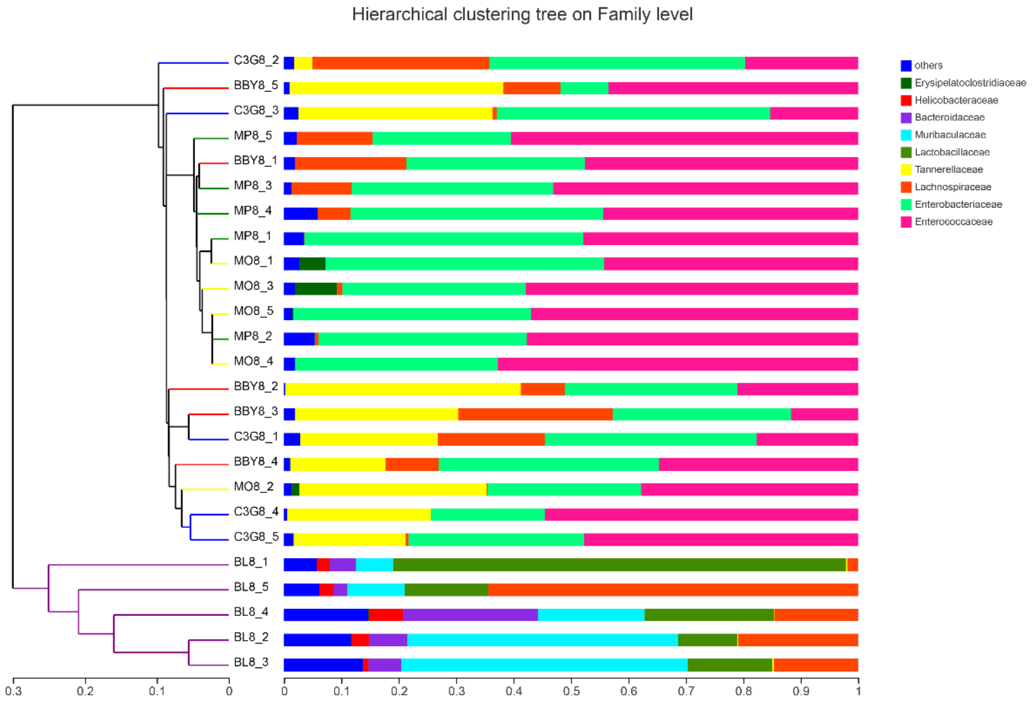

Supplementary Figure 7. Hierarchical clustering tree on Family level of stool samples in day 8.
